# CAROLS: A New Airborne L-Band Radiometer for Ocean Surface and Land Observations

**DOI:** 10.3390/s110100719

**Published:** 2011-01-12

**Authors:** Mehrez Zribi, Mickael Pardé, Jacquline Boutin, Pascal Fanise, Daniele Hauser, Monique Dechambre, Yann Kerr, Marion Leduc-Leballeur, Gilles Reverdin, Niels Skou, Sten Søbjærg, Clement Albergel, Jean Christophe Calvet, Jean Pierre Wigneron, Ernesto Lopez-Baeza, Antonio Rius, Joseph Tenerelli

**Affiliations:** 1 CESBIO (CNRS/IRD/CNES/UPS), 18 avenue Edouard Belin, 31401 Toulouse Cedex 9, France; E-Mail: yann.kerr@cesbio.cnes.fr; 2 Université Versailles St-Quentin, UPMC, CNRS/INSU, LATMOS-IPSL (UVSQ/CNRS/UPMC), 11 Boulevard d’Alembert, 78280 Guyancourt, France; E-Mails: parde@latmos.ipsl.fr (M.P.); pascal.fanise@latmos.ipsl.fr (P.F.); hauser@latmos.ipsl.fr (D.H.); monique.dechambre@latmos.ipsl.fr (M.D.); mll@latmos.ipsl.fr (M.L.-L.); 3 LOCEAN, Tour 45-55-5eme étage, Paris, France; E-Mails: jacqueline.boutin@locean-ipsl.upmc.fr (J.B.); reve@locean-ipsl.upmc.fr (G.R.); 4 DTU-Space, National Space Institute, DTU, B. 348, DK 2800, Lyngby, Denmark; E-Mails: ns@space.dtu.dk (N.S.); sss@space.dtu.dk (S.S.); 5 CNRM/GAME (Météo-France, CNRS), URA 1357, Toulouse, France; E-Mails: clement.albergel@meteo.fr (C.A.); jean-christophe.calvet@meteo.fr (J.C.C.); 6 INRA, UR1263 EPHYSE, F-33140 Villenave d'Ornon, France; E-Mail: jpwigner@bordeaux.inra.fr; 7 Facultat de Fisica, Universitat de Valencia, C/Dr Moliner, 50, Burjassot, 46100 Valencia, Spain; E-Mail: Ernesto.Lopez@uv.es; 8 IEEC/ICE-CSIC, Campus UAB/Fac Ciencies, Torre C-5-parell-2^a^ planta, 08193 Bellaterra, Spain; E-Mail: rius@aliga.ieec.uab.es; 9 National Institute of Marine Research (CLS), Brest, France; E-Mail: jtenerelli@cls.fr

**Keywords:** radiometer, CAROLS, L band, SMOS, ocean salinity, soil moisture

## Abstract

The “Cooperative Airborne Radiometer for Ocean and Land Studies” (CAROLS) L-Band radiometer was designed and built as a copy of the EMIRAD II radiometer constructed by the Technical University of Denmark team. It is a fully polarimetric and direct sampling correlation radiometer. It is installed on board a dedicated French ATR42 research aircraft, in conjunction with other airborne instruments (C-Band scatterometer—STORM, the GOLD-RTR GPS system, the infrared CIMEL radiometer and a visible wavelength camera). Following initial laboratory qualifications, three airborne campaigns involving 21 flights were carried out over South West France, the Valencia site and the Bay of Biscay (Atlantic Ocean) in 2007, 2008 and 2009, in coordination with *in situ* field campaigns. In order to validate the CAROLS data, various aircraft flight patterns and maneuvers were implemented, including straight horizontal flights, circular flights, wing and nose wags over the ocean. Analysis of the first two campaigns in 2007 and 2008 leads us to improve the CAROLS radiometer regarding isolation between channels and filter bandwidth. After implementation of these improvements, results show that the instrument is conforming to specification and is a useful tool for Soil Moisture and Ocean Salinity (SMOS) satellite validation as well as for specific studies on surface soil moisture or ocean salinity.

## 1. Introduction

Passive microwave remote sensing of Soil Moisture (SM) and Sea Surface Salinity (SSS) has been at the center of attention of many research programs for several decades. Various airborne and *in situ* radiometers have been developed, showing the high potential of L-band measurements for the estimation of surface parameters [1–6].

The SMOS satellite mission is based on an aperture synthesis L-band radiometer [7,8], designed and developed by the European Space Agency (ESA). In the context of the validation activity for the SMOS mission, the authors proposed to design, build and operate the CAROLS L-Band radiometer from an aircraft. Because the sensitivity of L-band brightness temperature to salinity is very small (−0.45 K/psu at a physical temperature equal to 105 K), it was necessary to build a very accurate, sensitive, and stable system. The radiometer was installed in the French research ATR-42 aircraft. In coordination with *in situ* field campaigns, it is used together with a suite of other airborne instruments: a scatterometer—or a C-Band radar—used to measure ocean roughness and winds, a GPS receiver to estimate ocean surface roughness, an infrared radiometer to measure sea surface temperature, and a visible wavelength wide angle camera to provide a qualitative description of the surface.

The first campaign was conducted in September 2007, for the qualification and certification of the aforementioned instruments. Following various improvements to the CAROLS instrument, a second campaign was carried out in November 2008, in order to validate the CAROLS data quality. In spring 2009, a scientific campaign was organized, to acquire different types of brightness measurements over oceanic and land surfaces. The main objective of this third campaign was to validate the direct and inversion models used to estimate Soil moisture and Sea Surface Salinity.

This paper presents a detailed description of the CAROLS instrument, of its refined calibration system, and of the first results obtained from the campaigns carried out in 2007, 2008 and 2009.

In Section 2, we present the instruments used in our campaigns, in particular the CAROLS radiometer, and in Section 3 we describe the campaigns. The CAROLS data quality is discussed in Section 4. Our conclusions are given in Section 5.

## 2. Airborne Instruments and Interface with the ATR-42

### 2.1. CAROLS Radiometer

#### 2.1.1. System Description

##### 2.1.1.1. General System Design

CAROLS is a total power radiometer and has a simple structure and high theoretical sensitivity. The receiver was developed as a copy of the EMIRADII radiometer [9,10], in collaboration between the TUD (Technical University of Denmark) and the LATMOS laboratory (Laboratoire Atmosphères, Milieux, Observations Spatiales). It is a fully polarimetric correlation radiometer using direct sampling [9,10]. The antenna system comprises two large, identical Potter horns and two waveguide orthomode transducers (OMT). These provide dual–incidence measurements, useful for the estimation of soil moisture or ocean salinity from brightness temperatures.

The CAROLS system periodically performs two-point calibrations, to calibrate the receiver gain and noise temperature by means of noise injection. In such a calibration system, the receiver looks at known sources and makes use of their associated outputs to infer the relationship between the output value provided by the A/D converter and the antenna temperature. A matched load is coupled with a noise source, which can be switched on and off, in order to measure two different brightness temperatures. Laboratory calibration of the instrument consists in determining a set of parameters relating the power received from the noise source to its physical temperature.

All of the analog components are placed inside a double insulated and thermally regulated box and their physical temperatures are recorded in real time by means of temperature sensors. The accuracy of the receiver is highly dependent on the stability of the noise source and other components used in the noise injection circuitry.

##### 2.1.1.2. Technical Specifications

The main technical specifications are presented in the following table (Table 1) where AFE and DFE refer to the ‘Analog Front End’ and ‘Digital Front End’ electronics respectively:

##### 2.1.1.3. Detailed System Design

###### A. Receiver Unit

####### a. Analog Front End (AFE) Electronics

**Figure 1.**
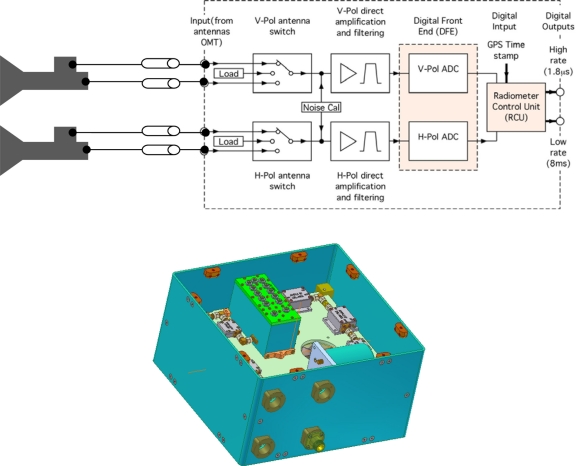
Block diagram of the antenna and receiver unit.

The Analog Front End (AFE) is composed of several microwave components (switch, director coupler, noise source, filter, isolator). The antenna and receiver unit is illustrated in Figure 1.

This unit ensures a gain of 81 dB for each channel and an isolation of approximately 80 dB between the H (Horizontal) and V (Vertical) channels. Special attention was paid to the performance of the bandpass filter design. This device, based on cavity properties, provides a center frequency of 1,413 MHz and a 3 dB bandwidth of 24 MHz. The filter was specially tuned to attenuate interfering signals lying outside the bandwidth of interest.

The performance of the analog front end is strongly dependent on its physical temperature. Therefore, to achieve the desired radiometric sensitivity, the measurement of inside temperatures is very important for the calibration process. The receiver is fitted with approximately ten thermal sensors, placed at various locations. The microwave section is thermally controlled using a PI (Proportional Integral) regulation algorithm. The AFE demonstrated its excellent ability to stabilize the internal temperature at 45 degree: during a 4-hour flight this varies by only 0.1 degree.

####### b. Digital Front End (DFE) Electronics

The analog signal output from the analog receiver is directly digitized by two A/D converters, with a sampling frequency of 139.4 MHz. The unit is designed for sub-harmonic sampling of the analog signals, *i.e.*, direct sampling of the L-band signal with no analog down conversion. The signals produced by the converter are fed into a FPGA (Field Programmable Gate Array), in which 1.8 μs and 1 ms integrations are computed. The 1.8 *μ*S integration period is useful during post processing, for the removal of Radio Frequency Interferences (RFI).

###### B. Antenna Unit

The antenna unit comprises a horn and an orthomode transducer (OMT). Each horn has an identical pattern and a 37.6° half-power beam width (HPBW), for both horizontal and vertical polarizations. This large beam and corresponding antenna dimensions were specified in order to fit the lower fuselage apertures of the aircraft. The antenna system (OMT and Potter horns) is characterized by almost ideal radiation patterns with practically no side-lobes and low losses. The performance of the Potter horns was tested at the CNES’ (Centre National d’Etudes Spatiales) anechoic chamber, and has been judged to be very satisfactory, with its side and back lobes being suppressed by more than 30 dB, and an even lower cross-polarization level. The performance of each OMT was also measured. Isolation between ports was greater than 49 dB for all frequencies between 1.4 and 1.427 GHz.

###### C. Antenna and Cable Losses

**Figure 2.**
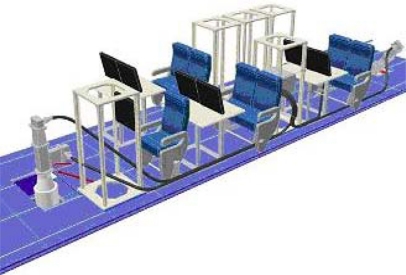
The CAROLS radiometer inside the ATR42 research aircraft, **(a)** illustration of nadir antenna inside the aircraft, **(b)** illustration of CAROLS system (receiver and antennas) inside the aircraft.

Each attenuator not only attenuates the incoming signal, but also introduces an associated thermal noise [1]. In the aircraft, each antenna was insulated to minimize temperature variations within its structure. Indeed, it was our aim to maintain identical temperatures at each port. The temperature of each OMT and horn was continuously monitored by means of a set of thermistors. Because coaxial cable losses are the main sources of signal error, a significant effort was made to very accurately determine its influence. Four 4.5 m long cables are needed to connect each antenna to the receiver unit, as shown in Figure 2. These cables transport signals (corresponding to the vertical and horizontal polarizations), which require predictable insertion phase and insertion loss characteristics. For the present application, it was decided to use SUCOFEED 1¼ coaxial cable from HuberSuhner, which has very low losses (0.19 at 30 °C) and a very low drift coefficient (0.055 K/C). Because the temperature in the aircraft cabin is not stabilized, the coaxial cables are insulated and equipped with five temperature sensors mounted inside their insulating cladding. Insertion phase is also a very important characteristic for RF cables. Although phase matched cables are not mandatory for radiometric applications, phase-stabilized cables must be used. The relative phase matching requirement is satisfied by matching the phase, as opposed to the absolute length, of the two cables linked to each antenna relative to each other.

#### 2.1.2. Performance of the Instrument

##### 2.1.2.1. Sensitivity

**Figure 3.**
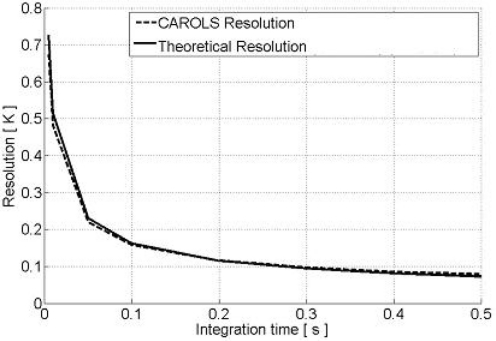
Radiometric resolution of the CAROLS radiometer.

The radiometric sensitivity (or radiometric resolution), referred to as *NEDT*, is the minimum detectable change in the radiometric antenna temperature of the observed scene. This sensitivity is directly related to the rapid fluctuations of the measured radiometric temperature. The radiometric sensitivity, related to the thermal noise *NEDT* of the radiometer, is given by:

(2) $$\mathrm{NEDT}=\frac{T_{\mathrm{in}}+T_{R}}{\sqrt{\tau B}}$$

where *T_in_* is the temperature at the input of the receiver, *T_R_* is the noise temperature of the receiver, τ the integration time and *B* the bandwidth of the receiver. In Figure 3, we illustrate the theoretical sensitivity and the CAROLS sensitivity measured in the laboratory, as a function of integration time. It can be seen that for an integration time longer than 300 ms, the sensitivity is better than 0.1 K.

##### 2.1.2.2. Linearity

**Figure 4.**
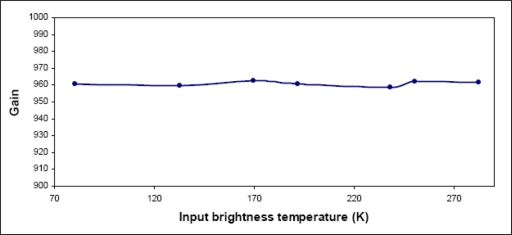
Illustration of CAROLS receiver linearity.

The linearity of the radiometer receiver, over a range of temperature covering natural ocean and land emissions, is crucial to obtain consistent data. A proven technique for linearity validation involves the use of a cryogenic load providing a very cold brightness temperature at the input, a variable attenuator, and a data collection system at the system output. For each attenuation value, the receiver output is determined with the noise diode being switched on and off. This experiment is run when the system is correctly regulated, and it is assumed that the noise diode adds a constant noise, at the receiver input. The linearity measurements indicate worst-case scale variations of the order of 0.4% (Figure 4).

##### 2.1.2.3. Stability

**Figure 5.**
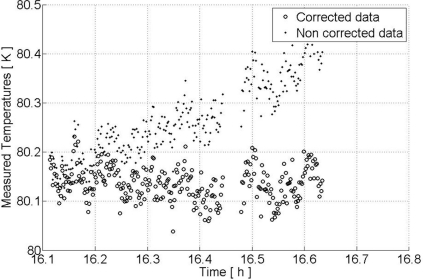
Stability of the CAROLS measurements. Non corrected data correspond to one calibration point; corrected data correspond to two calibration points before and after data acquisition.

Figure 5 provides two plots of calibrated temperatures, measured by CAROLS in the laboratory, when exposed to a target simulated by a cryogenic load (with a stable temperature of 80.1 K). The black points correspond to measurements calibrated using only one initial calibration period, before the cryogenic load measurement, whereas the circles indicate measurements calibrated using two calibration times (before and after the cryogenic measurements), separated by an interval of 30 minutes. For each calibration, the gain and the receiver noise temperature are estimated with help of two calibrated points (load and load + noise diode). Using two calibration phases, the measured gain and receiver noise temperature are interpolated. We show that, whereas for the first calibration point only, if the temperature drift is approximately 0.1 K (between 16.1 H and 16.4 H), after 20 mn, the use of two calibration times leads to a variation in measured temperature of less than 0.08 K, after 30 mn.

### 2.2. Auxiliary Airborne Instruments

**STORM Radar:** STORM, developed at LATMOS [11,12], is a C-Band radar (5.35 GHz) based on an FM/CW (Frequency-Modulated/Continuous Wave) transmitted waveform. It transmits alternatively in the H and V polarizations, and simultaneously receives in both of these channels. The antenna is mounted so as to point towards the ground with a 20° incidence angle (measured with respect to nadir), when the aircraft’s attitude is horizontal. STORM data are acquired over the ocean to provide information concerning the wind and waves, to provide complementary data to the CAROLS radiometric measurements, which are sensitive to wind, sea surface temperature and sea surface salinity.

**GPS GOLD-RTR System:** The GPS system was supplied by the IEEC (Institut d'Estudis Espacials de Catalunya or Institute for Space Studies of Catalonia) [13,14]. It is composed of three antennas and a GPS Open Loop Differential Real Time Receiver (GOLD-RT). The aim is to retrieve information concerning sea surface roughness, in the form of the effective Probability Density Function (PDF) of the sea surface slopes.

**Infrared Radiometer (IRR):** The infrared radiometer is part of the standard equipment of the research ATR42. It is used to provide surface temperature estimations, simultaneously with the CAROLS measurements.

**Visible Wide Angle Camera (VWAC):** It is located slightly forward of the slant radiometer antenna and allows the areas covered by the CAROLS nadir antenna measurements to be identified.

### 2.3. Description of the Interfaces with the Research ATR-42 Aircraft

**Figure 6.**
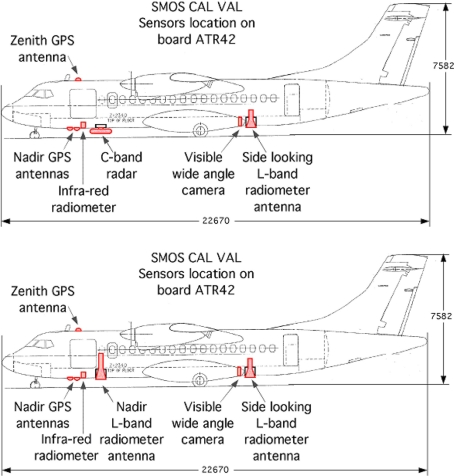
**a.** (top) CAROLS instrument with one slant antenna and the STORM instrument, **b.** (bottom) CAROLS instrument with two antennas (one slant and one nadir).

The research ATR42 has been available for research experiments since early 2006 (http://www.safire.fr). It is an original ATR42-320, specifically modified for scientific use. Because of limitations in terms of the existing lower fuselage apertures in the aircraft, *i.e.*, only two openings (one forward aperture: 650 × 450; one aft aperture: 700 × 450 mm), two configurations were proposed for the CAROLS campaign flights, as shown in Figure 6. In the first configuration, providing dual-incidence data, the CAROLS radiometer was operated with two antennas, one pointing to nadir for vertical measurements, and the other pointing to the right side of the aircraft (slant antenna), at an approximately 30° incidence angle. The second configuration combines the CAROLS slant antenna, placed at the aft of the aircraft, with the STORM instrument placed at the front (at the location of the nadir antenna used in the first configuration). This second configuration is particularly useful for ocean measurements. The true incidence angles corresponding to each brightness temperature measurement were calculated accounting for the attitude of the aircraft (roll, pitch), with an accuracy of 0.1 degrees.

## 3. Description of the Carols Campaigns

### 3.1. Flight Descriptions

The CAROLS radiometer was operated with the two configurations described above (with one or two antennas), during all of the CAROLS flights. Table 2 provides details of the three CAROLS campaign flights. All measurements were made from altitudes ranging between 600 m and 3,000 m. Different flight patterns were carried out during the oceanic flights, in addition to straight horizontal flight portions: wing wags with a ±25° roll; nose wags with a ±5° pitch; and circular flights with 15° positive and negative roll angles. These movements are useful for the qualification of the instrument’s behavior and validation. The wing wags provide measurements over a large range of incidence angles, which are particularly useful for the validation of the emissivity models. They allow inter-comparisons between measurements recorded with the two antennas, thus making it possible to validate the loss term estimations and antenna alignment. Finally, they also allow the two polarization channels to be phase matched. The circular flights were defined in order to measure the full azimuthal variation of the Stokes vector, within the shortest possible time.

### 3.2. Measurement Sites

We selected three different test sites for the CAROLS data acquisitions, over land and ocean surfaces. Simultaneously to the CAROLS measurements, different *in situ* measurements were acquired in order to qualify the CAROLS data, the direct emissivity models, and finally the inversion algorithms.

**Figure 7.**
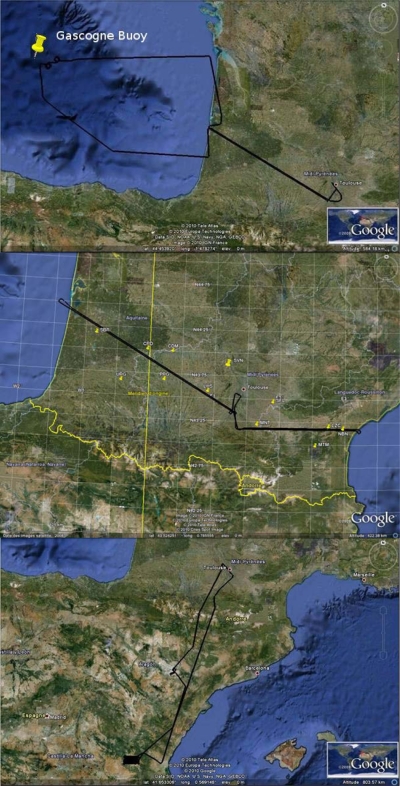
Illustration of flight transects, **a** (top) illustration of one ocean flight transect over the Gulf of Biscay, **b** (middle) Illustration of a SMOSMONIA flight transect. Markers are pointing to the location of the 12 SMOSMANIA measurement sites, **c** (bottom) Illustration of a Valencia transect flight.

The ocean measurements were made over the Bay of Biscay [Figure 7(a)]. In order to support the Airborne CAROLS experiment, ship campaigns were organized by the LOCEAN team for each CAROLS campaign, along the same track as that used by the aircraft. Measurements of SSS, sea surface temperature (SST), wind speed, foam, wave spectrum, heat and momentum flux were collected on the ship. Different drifters measuring SSS and SST were deployed. All flights passed over or close to the Gascogne Buoy (45.2°N, 5°W), which is an operational buoy operated by the French Meteorological Office providing SST, pressure, wind, wave spectrum.

This first land site, characterized by a temperate climate, is situated in the South West of France [Figure 7(b)]. The Soil Moisture Observing System—Meteorological Automatic Network Integrated Application (SMOSMANIA) determines soil moisture values, based on a portion of automatic ground station network (the RADOME network) [15].

The second land Site, with semi-arid climate, is located near Valencia in South East Spain, about 80 km inland, to the West of Valencia [Figure 7(c)]. Within the Valencia validation site, an area of 30 km × 50 km was selected for the experiment [16].

## 4. Carols Airborne Data Analysis

### 4.1. Methods for Qualification of CAROLS Data

After qualification of the CAROLS radiometer in the laboratory, the analysis of our CAROLS data was based mainly on comparisons between the brightness temperatures measured over the ocean (in X and Y channels) and simulated brightness temperatures. *Tx* and *Ty* brightness temperatures are defined as:

$$\begin{array}{l} \mathrm{Tx}=\frac{I+Q}{2}\\ \mathrm{Ty}=\frac{I-Q}{2} \end{array}$$

where I and Q are the first and second Stockes parameters.

For the purposes of this comparison, we adapted the Terrestrial Radiometry Analysis Package (TRAP) software originally developed by IFREMER for the analysis of COSMOS campaign measurements [17].

#### 4.1.1. TRAP Software Tool

The TRAP software allows the various contributions affecting the L-band brightness temperatures to be simulated. It uses the physical modeling of celestial, atmospheric and oceanic signals as for the processing of SMOS salinity data [17]: the atmospheric emissivity and absorption model from Liebe e*t al.* [18]; the sea surface emissivity for a flat sea surface from Klein and Swift [19]; in our analysis, by default, the contribution to emissivity of the sea surface roughness is estimated using a two-scale model based on the Durden and Vesecky x2 wave spectrum [20] (2scale/DV2); we also tested the rough surface emissivity simulated by a small slope approximation model, and the Kudryatsev *et al.* [21] wave spectrum (SSA/Kudr.). Scattering of galactic noise by the ocean surface has been simulated by Tenerelli *et al.* [22]. The atmospheric pressure and relative humidity data needed to run the atmospheric model were taken either from the Gascogne buoy (45.2°N, 5°W), or from the European Center for Medium range Weather Forecast (ECMWF); the wind speed needed to estimate the impact of roughness on the sea surface emissivity, and on the sea surface scattering of atmospheric and galactic signals, was taken from QuikSCAT measurements recorded within less than two hours of the CAROLS flights, or from the Gascogne buoy measurements. The sea surface salinity and temperature values needed to simulate the sea surface emissivity were derived from the ship and buoy measurements. Our primary goal was to validate the instrument. Thus, in order to achieve a clear separation between the analyses of data recorded by each antenna port, we chose to simulate the antenna temperatures in the polarization reference system defined by the antenna framework. This allowed us to avoid mixing the measurements made by different antenna ports, which would be needed to reconstruct horizontally and vertically polarized signals. TRAP takes on account aircraft maneuvers to consider mixing between polarizations, for each antenna port. Integration of the simulated signal over the gain of the antenna pattern was achieved using the antenna diagram measured in the laboratory (very close to a Potter horn).

#### 4.1.2. CAROLS Calibration Data

During any given flight, periodic calibrations effectively decrease the influence of gain fluctuations, and improve the radiometer’s sensitivity. Internal calibration consists in switching the receiver input between an internal load and a noise diode with the power turned alternately off and on. During campaigns, it is very important to monitor the drift of each instrument. The measured gain and receiver noise temperature are interpolated between successive calibration phases, in order to estimate the receiver’s behavior during the antenna measurement, and to apply suitable corrections to the recorded values.

In order to retrieve absolute values of natural emissions, absolute calibration of the measured brightness temperatures is needed. The absolute calibration of the radiometers is generally made by measuring the antenna temperatures while flying over fresh water lakes. In our case, this type of measurement was not possible because of the persistent presence of strong RFI around identified lakes in France. For this reason, absolute calibrations were carried out over the ocean. For the two antennas, the measurements were compared to calculated temperatures using the TRAP software. For these comparisons, measurements recorded under conditions of only limited variations in roll and pitch, and also SSS, were used.

### 4.2. CAROLS Results

#### 4.2.1. CAROLS 2007 Campaign

##### 4.2.1.1. Ocean Measurements

**Figure 8.**
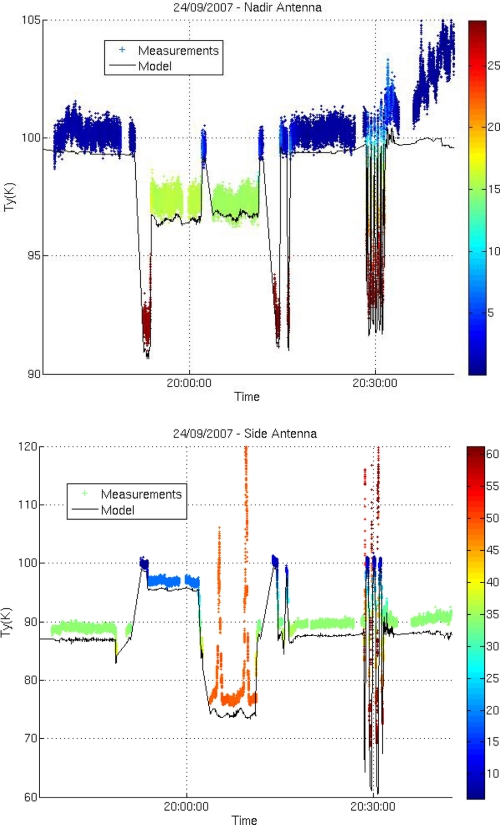
Measurements (8 ms average) recorded at the Y port (close to H-pol), by the nadir (top) and side (bottom) antennas on 24 September 2007. Incidence angles are indicated by the color coding. The simulated values of Ty are indicated by the black line.

Figure 8 compares simulated and measured brightness temperatures acquired over the Gulf of Biscay. On the whole, in spite of the systematic bias between simulations and observations, there is a good agreement between the variations of the measured and simulated values of Ty. The roll and pitch movements of the aircraft allow a wide range of incidence angles to be explored.

Large fluctuations observed in Figure 8 denote radio-frequency interference effects occurring on the side-antenna during circles and wing wags at large incidence angles, when the antenna was pointing towards the south-east, and on both antennas at the end of the flight when approaching the coast; a bias is observed between measurements and simulations, throughout all flights. Investigations after the flights revealed that the radiometer bandwidth was too large, and that a connector was imperfectly isolated. These flaws were corrected for the 2008 and 2009 flights.

**Figure 9.**
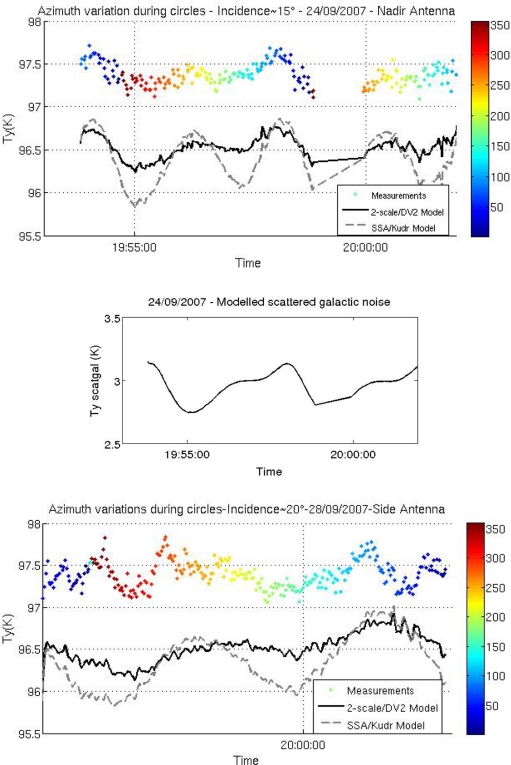

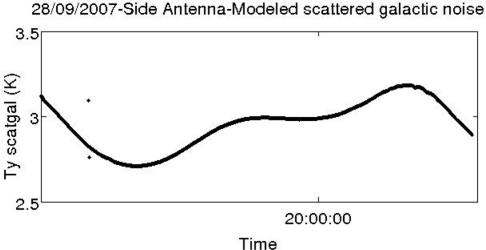
Top: Variations in Ty (1s averages) as observed (colored points), and simulated using the 2scale/DV2 (black) and SSA/Kudr. (grey) models during three circular flights. The azimuth angle is color coded. Bottom: Modeled variations of scattered galactic noise. Left: nadir antenna on 24/09/07; wind speed of 8.3 m/s. Right: side antenna on 28/09/07; wind speed of 8.4 m/s. (a bias has been artificially added to the measurements, to facilitate visual interpretation).

Circle flights allowed a wide range of azimuth angles to be explored. Figure 9 illustrates the azimuthal variation of the signal observed and simulated with both antennas, when the incidence angle is close to 15°. By separating in the simulation each component of the scattered signal, we would check that most of the observed azimuthal variation is due to surface scattering of galactic noise. At these incidence angles, the region of the sky affecting the measurements includes the Cygnus A source and part of the Milky Way, leading to ∼0.5 K azimuthal variations in the scattered galactic signal (see Figure 9, bottom). Furthermore, the simulations show (Figure 9 top) that the 2scale/DV2 model is consistent with the observations, whereas the second harmonic of Ty predicted by the SSA/Kudr. model appears to be too large.

**Figure 10.**
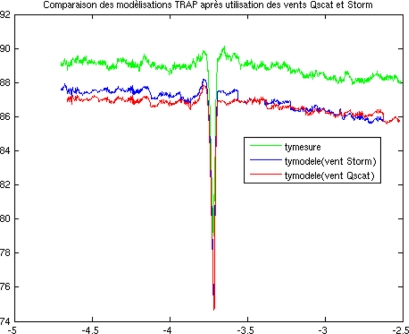
Variations in Ty (1 s averages) are observed (green) and simulated using either QSCAT wind speed values (red), or wind speed values derived from the STORM scatterometer (blue) on 28/11/07, along latitude 45.5°N. The low values observed at 3.7°W correspond to a change in direction of the aircraft.

On 28 September 2007, the wind speed increased from 3 to 9 m/s, from east to west along latitude 45.5°N. A 1K east-west gradient of Ty was observed during the northern flight on 28 September. This was associated with a 6 m/s wind speed gradient and with a 0.5 pss salinity gradient between the longitudes 2.7°W and 4.7°W. Simulations with the 2-scale emissivity model and DV2 spectrum are consistent with the observed gradient (see Figure 10).

In this particular case, close to the longitude 3.5°W, it can be seen that the STORM derived wind speeds were helpful in interpreting the observed variability; indeed, contrary to the QSCAT wind speeds, the STORM measurements were acquired at the same time as the CAROLS data, and have a better spatial resolution (about 6 km × 4 km instead of 25 km × 25 km for Quickscat).

##### 4.2.1.2. Continental Measurements

**Figure 11.**
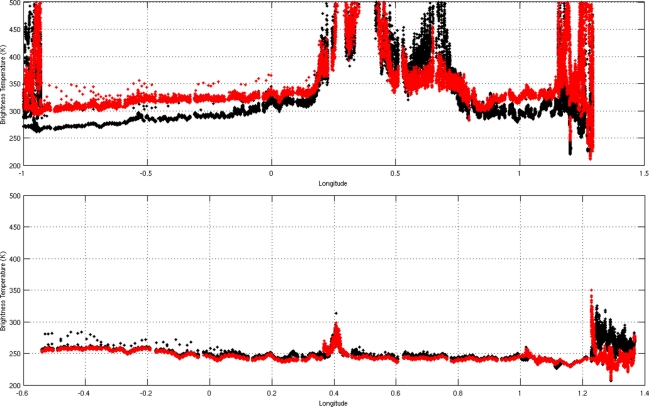
Illustration of the data acquired during two CAROLS flights over the same transect, using the slant antenna, **a** (top) a flight in 2007, affected by strong RFI signals over land surfaces, **b** (bottom) a flight in 2008, after changes to the filter, showing a significant decrease in the percentage of RFI signals over land surfaces. The red points indicate the X polarization values, whereas the black ones indicate the Y polarization values (nearly corresponding to the V and H polarizations.

Figure 11 illustrates the brightness measurements recorded over South West France on September 2007 (a) and November 2008 (b), with the nadir antenna, for both horizontal and vertical polarizations. We note the strong presence of two types of RFI, especially in 2007: the first of these was a particularly strong continuous noise signal characterized by very high temperature values, detected mainly in two areas: around the city of Auch (longitude approximately equal to 0.4°E) and close to the CEL (Centre d’Essai des Landes) area in South West France (longitude approximately equal to 0.4°W). A second type of signal was composed of periodic pulses, probably due to radars. Types and mitigation of RFI are discussed in [25]. Outside the zones affected by RFI, the brightness temperature level measured by the Y port is qualitatively consistent with the *in situ* ground moisture measurements.

##### 4.2.1.3. Conclusions

In conclusion, the CAROLS data shows a good level of agreement with the simulations, illustrating in particular its excellent sensitivity. These encouraging results were achieved despite the identification of an imperfect isolation between the two polarization channels. This problem led to errors in the estimation of *Tx*, and therefore to errors in the third and fourth Stokes parameters. Secondly, we observed a very strong presence of RFI over continental surfaces.

#### 4.2.2. CAROLS’2008 Campaign

The aim of the second campaign (CAROLS’2008) was to validate the modifications implemented in order to correct the two technical faults identified during the initial CAROLS’2007 campaign. The first modification enabled the X and Y channels to be completely isolated.

The second technical problem was related to the filter bandwidth. In fact, following a ground search for possible RFI sources in the studied region, we identified the sources responsible for most of the RFI. These correspond to antennas with an emission frequency very close to our protected band. For this reason, in 2008 the filter bandwidth was decreased from 26 MHz (1,400–1,426 MHz) to 24 MHz (1,401–1,425 MHz) at −3dB, thus making it more selective.

Figure 11 illustrates the extent of RFI detected over South West France, before and after this modification to the filter bandwidth. Although some RFI was still present during the 2008 measurements, its level was significantly reduced. In order to correct the data for this noise, different methodologies were applied, based in particular on the Kurtosis parameter, or other filters which apply thresholds based on the standard deviation of Stokes parameter variations [23–25]. These approaches allowed, for example, less than 30% of the measurements over the SMOSMANIA site to be flagged during the CAROLS 2008 flights.

#### 4.2.3. CAROLS 2009 Campaign

The first two campaigns allowed the CAROLS radiometer data to be qualified. The main objective of the CAROLS’2009 campaign was to acquire scientific data, to improve direct and inversion models of land and ocean surfaces. Following the data calibration steps described in the above sections, we retrieved the four Stokes parameters (I, Q, U and V) with respect to the antenna reference frame.

##### 4.2.3.1. Ocean Measurements

**Figure 12.**
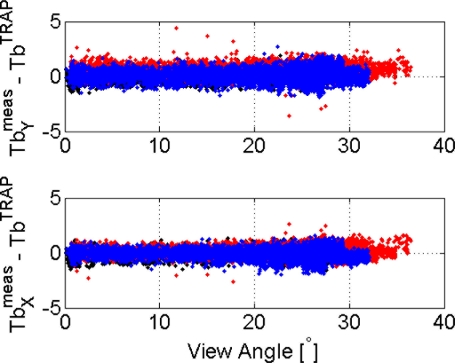
Comparison between measured and simulated antenna temperatures (in K)—for the Y (top) and X (bottom) polarizations, with the nadir antenna—as a function of view angle during the wing wag movements, for the three different flights (black: the first flight, red: the second flight, blue: the third flight) over the ocean during the CAROLS’2009 campaign.

Figure 12 shows the computed differences between the TRAP software simulations and the measured *Tx* and *Ty* values during the wing wag measurements. The mean differences are found to be less than 1 K for all flights, for the measurements sampled at 40 ms (with large std values).

**Figure 13.**
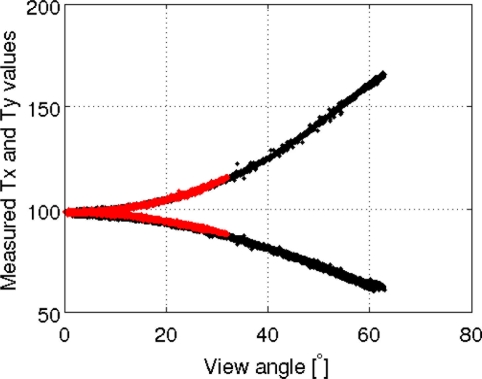
Tb (K) measured for the X and Y polarizations, for both nadir (red) and slant (black) antennas, as a function of incidence angle.

Figure 13 shows the data collected over the ocean, influenced by wing wag movements with strongly varying incidence angles, for both antennas. The results indicate a strong degree of consistency between the data collected by the two antennas. The upper Tb values are for the X polarization, whereas the lower values correspond to the Y polarization. The nadir antenna data are plotted in red, and that with the slant antenna is plotted in black. For the same incidence angle, the variations between the nadir and slant antenna measurements are less than 0.2 K for the X polarization and less than 1 K for the Y polarization.

In the case of the third and fourth two Stokes parameters (T3 and T4), the analysis must take into account the fact that the cables were not completely phase matched as a result of their long length. A correction was thus applied to eliminate the influence of this effect on the correlation between the horizontal and vertical measurements. Computation of the phase difference angle is based on the comparison of recordings made during wing wag movements, which enabled an angle of approximately 42° to be determined.

**Figure 14.**
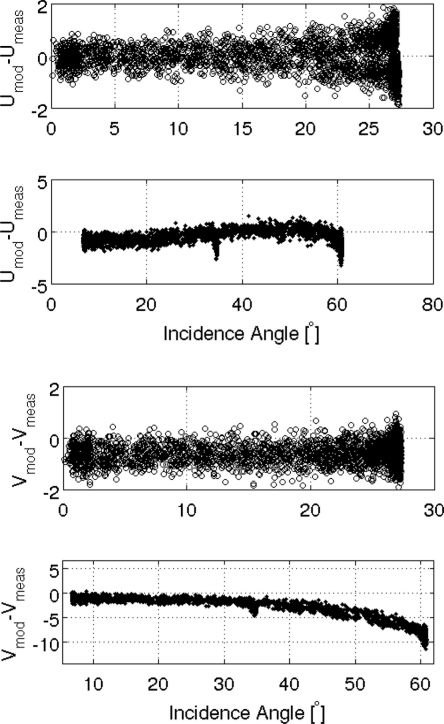
T3 and T4 differences between modeled and measures values (Gulf of Biscay) during the 2009 CAROLS flights, for ±25° wing wags: nadir antenna (upper figures); slant antenna (lower figures).

Figure 14 provides a comparison between the corrected measurements and the modeled third and fourth Stokes parameters (using TRAP software). For the third parameter, a good agreement is observed: the CAROLS measurements fit the model estimations for all incidence angles, with variations of less than 2 K. In the case of the fourth Stokes parameter, although we find a good agreement for low incidence angles with nadir antenna, small but increasing discrepancies are found as the incidence angle increases; these differences reach a value of −10 K with slant antenna. This is probably due to simulation errors resulting from the large antenna beam width, reaching very high angles at an incidence greater than 50°.

##### 4.2.3.2. Continental Measurements

**Figure 15.**
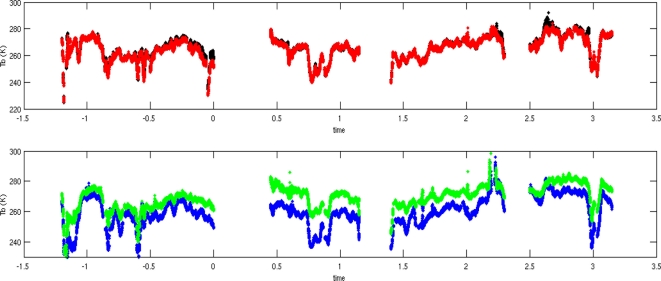
Time variations of Tb during the SMOSMANIA transect, in 2009, for the nadir antenna (upper figure, red and black lines respectively for the X and Y polarizations), and slant antenna (lower figure, green and blue lines respectively for the X and Y polarizations). A mixed Kurtosis and thresholds to the Tb standard deviation algorithm [25] was applied for the data used here.

Figure 15 shows the measured temperatures recorded over part of the SMOSMANIA site, acquired with both the nadir and slant antennas after RFI elimination [25]. For the nadir antenna, we find a good agreement between the *Tx* and *Ty* measurements. For the slant antenna, we verify that the *Tx* (close to TV) values are higher than the *Ty* (close to TH) values. For all measurements, we qualitatively validate the agreement between brightness temperatures and ground moisture measurements, with an increasing value of Tb on the driest flight days (for SM variations of about 1%, we observed Tb variations of about 1.5 K). The detailed inversion results will be published in the near future.

## 5. Conclusions

The CAROLS L-Band radiometric instrument has been built and tested in the context of the SMOS mission. It will be an essential tool for SMOS calibration operations and for the improvement of direct and inverse modeling. The CAROLS radiometer has been validated and qualified with laboratory measurements, showing in particular excellent stability and resolution. The measurements demonstrated that this radiometer has a sensitivity of 0.1 K for a 1 s integration time, and a relative stability of 0.1 K over a period of 30 min. Three campaigns were then carried out. The first four flights made in September 2007 allowed CAROLS, installed in conjunction with other airborne instruments (STORM radar, GOLD-RTR GPS receiver, an infra-red radiometer and a visible wide angle camera), to be certified for use in the French research ATR-42 aircraft. The airborne measurements verified the good sensitivity of the CAROLS data, and its good agreement with the emissivity model during wing wags and circular movements of the aircraft. However, these initial flights allowed an imperfect isolation to be identified between the X and Y channels, and a high level of detected RFI to be identified over land surfaces. The aim of the CAROLS’2008 campaign was to validate various modifications, allowing the imperfections observed in 2007 to be corrected. Complete isolation of the two channels has been validated. Concerning the presence of RFI, our ground measurements identified the need to use a more selective frequency filter, in order to avoid high emission sources very close to our protected bandwidth (1,400–1,427 MHz). The new filter used during the 2008 flights permitted this RFI to be strongly attenuated in the data recorded over South West France. Finally, following validation of the CAROLS data recorded in 2007 and 2008, the initial objective of the 2009 CAROLS campaign was to acquire scientific data for the inversion algorithm, used to provide soil moisture and ocean salinity estimations. The retrieved first and second Stokes parameters are found to be in good agreement with the simulated values. All comparisons over the ocean show an absolute accuracy <1 K. The third and fourth Stokes parameters indicate a good degree of coherence with the simulated values, for low and medium incidence angles.

These measurement campaigns have been successful, and the detailed science results will be published in the near future. The next step will be the analysis of a major experimental campaign held in the spring of 2010, with the aim of validating the SMOS measurements.

## Figures and Tables

**Table 1.** Main technical specifications of the CAROLS radiometer.

CAROLS: correlation radiometer with direct sampling	

Receiver type	Total power receiver
Operating frequency	[1.4–1.427 GHz]
Bandwidth	24 MHz@ −3 dB
Polarization	Polarimetric (4 stockes parameters: I, Q, U, V)
Sampling rate	139.4 MHz
Integration time	1 ms and 1.8 μs
Internal calibration	Load and noise diode
Sensitivity	0.1 K for 1 s integration time
Stability	0.1 K
System noise temperature	150 K
Physical temperature of AFE	45 °C
Physical temperature of DFE	90 °C

**Table 2.** Description of the flights undertaken during the CAROLS campaigns.

	**CAROLS’2007**	**CAROLS’2008**	**CAROLS’2009**
**Period**	24/09/2007–28/09/2007	12/11/2008–24/11/2008	23/04/2009–28/05/2009
**Number of flights**	4 flights	4 flights	13 flights
**Studied sites**	Gulf of Biscay, South West of France	Gulf of Biscay, South West of France	Gulf of Biscay, South West of France, Valencia site
**Departure**	Toulouse (France)	Toulouse (France)	Toulouse (France)
